# Clinical and Endoscopic Profile among Patients with Dyspepsia in Patients attending Tertiary Care Center: An Observational Study

**DOI:** 10.31729/jnma.v63i290.9216

**Published:** 2025-09-01

**Authors:** Abashesh Bhandari, Kritika Bhattarai, Ashish Acharya, Supri Raj Shrestha

**Affiliations:** 1 Department of Gastroenterology and Hepatology, Internal Medicine, Nepal Medical College Teaching Hospital, Attarkhel, Kathmandu, Nepal; 2 Nepal Medical College Teaching Hospital, Attarkhel, Kathmandu, Nepal; 3 Department of Community Medicine, Nepal Medical College Teaching Hospital, Attarkhel, Kathmandu, Nepal

**Keywords:** *dyspepsia*, *helicobacter pylori infection*, *Nepal*, *prevalence*, *rapid urease test*

## Abstract

**Introduction:**

Dyspepsia is a cluster of gastrointestinal symptoms related to feeding due to an organic cause or in the absence of any etiology. H. pylori infection is one of the most prevalent etiologies which is often detected using a rapid urease test. This study aimed to correlate the clinical-endoscopic profile in dyspeptic patients and find out the prevalence of H. pylori infection among those patients.

**Methods:**

This observational cross-sectional study was conducted from 1st August, 2024 to 31st January, 2025 in a tertiary care hospital in Nepal after obtaining ethical clearance from the Institutional Review Committee (Reference number: NMC-IRC/01-081/082). A total of 180 patients aged 18 years and above with dyspeptic symptoms for more than three months were included by total enumeration sampling. Data were collected using a structured proforma, and upper gastrointestinal endoscopy with rapid urease testing was performed. All collected data were entered and analysed using the Statistical Package for the Social Sciences version 16.0.

**Results:**

Of the 180 participants, 83 (46.11%) were male and 97 (53.89%) were female. Retrosternal burning was reported by 165 (91.67%) participants and upper abdominal pain by 118 (65.56%). Among the 180 participants, 81 (45.00%) reported current smoking and 67 (37.22%) reported alcohol consumption.

**Conclusions:**

This study highlights that a large proportion of dyspeptic patients were diagnosed with acid peptic disease, and a high prevalence of Helicobacter pylori infection was observed using rapid urease testing compared to previous studies.

## INTRODUCTION

Dyspepsia is defined as a cluster of upper gastrointestinal symptoms. Barbara, et al. suggested dyspepsia to be an episodic or persistent abdominal symptom often related to feeding, due to some underlying organic causes or in the absence of detectable causes which is termed as being functional dyspepsia.^1,2^ An upper esophagogastroduodenoscopy remains the mainstay investigation. The most recognizable criteria for functional dyspepsia is the Rome IV criteria.^3,4^ Helicobacter pylori *(H. pylori)* infection remains the most prevalent etiology with most patients being asymptomatic and only 10-20% developing the disease.^5^ *H. pylori* infection can be detected using noninvasive methods like serology, urea breath test and invasive methods like histology and rapid urease test. ^6^

This study aims to correlate the clinical and endoscopic profile in dyspeptic patients and to find out the prevalence of *H. pylori* infection induced peptic ulcer disease among those patients under the study.

## METHODS

This was a hospital-based observational cross-section study conducted from August 2024 to January 2025 among patients presenting with dyspeptic symptoms at the outpatient and inpatient departments of a tertiary care hospital in Nepal. The study aimed to determine the prevalence of Helicobacter pylori infection in this population.

Ethical clearance was obtained from the Institutional Review Committee (Reference number: NMC-IRC/01-081/082).

A total of 180 participants with dyspeptic symptoms were enrolled. Patients aged 18 years and above who presented with dyspeptic symptoms for more than three months, with symptom onset at least six months prior to diagnosis, and who underwent diagnostic upper gastrointestinal endoscopy were included.

The inclusion criteria were patients aged >18 years, diagnosed or admitted with dyspepsia, with or without treatment, and undergoing diagnostic upper gastrointestinal endoscopy. The patients who were younger than 18 years and those unfit for endoscopy were excluded.

Total enumeration sampling was employed. A total of 180 participants who met the eligibility criteria during the study period were included. The participants were further tallied based on the history they provided and the physical examination findings. Upper GI Endoscopy was performed among the participants to differentiate between functional dyspepsia and acid peptic disease. Further testing with rapid urease test was done to identify the number of individuals with H. pylori infection. The participants in the study were categorized into different age groups. The presence of ulcers, erosions, or inflammation in the stomach and duodenum observed during endoscopy was classified as Acid Peptic Disease (APD). The absence of these findings was classified as Functional Dyspepsia (FD).

A structured proforma, developed after expert review, was used to collect sociodemographic, clinical, and endoscopic information. Patient history and clinical findings were recorded in the same proforma.

All collected data were entered and analyzed using the Statistical Package for the Social Sciences version 16.0. Frequency and percentages were calculated for binary data.

## RESULTS

Out of a total of 180 participants in the study, 83 (46.11%) were male and 97 (53.89%) were female. A total of 94 (52.22%) participants were between 25 to 50 years of age (Table 1).

**Table 1:** Categorization of Participants Into Different Age Groups (n=180).

Age Group	n(%)
<25 years	38(21.11)
≥25 to <50 years	94(52.22)
≥50 years	48(26.67)
Total	180(100.00)

**Table 2:** Symptoms and its Duration Among Participants (n=180).

Symptom	n(%)
Retrosternal burning	165(91.67)
Upper abdominal pain	118(65.56)
Vomiting	33(18.33)
Early satiety	66(36.67)
Postprandial fullness	64(35.56)
**Duration of Symptoms**	
1-3 months	74(41.11)
≥3 months	106(58.89)
Total	180(100.00)

Out of a total of 180 participants enrolled in the study, 165 (91.67%) and 118 (65.56%) individuals presented with symptoms of retrosternal burning and upper abdominal pain, respectively. A total of 106 (58.89%) participants reported these symptoms for more than 3 months (Table 2).

Among the 180 participants, 81 (45.00%) reported current smoking and 67 (37.22%) reported alcohol consumption. A total of 14 (7.78%) participants reported using medications to relieve dyspeptic symptoms, and 7 (3.89%) reported medication use for concomitant psychiatric disorders.

Out of 115 (63.89%) endoscopically diagnosed cases of APD, 67 (36.89%) were male and 48 (26.67%) were female. Among the 65 (36.11%) cases classified as FD, 16 (8.89%) were male and 49 (27.22%) were female (Table 3).

**Table 3:** Cases of Acid Peptic Disease and Functional Dyspepsia After Esophagogastroduodenoscopy (n=180).

Diagnosis	Male n (%)	Female n (%)	Total n (%)
APD	67 (36.89)	48 (26.67)	115 (63.89)
FD	16 (8.89)	49 (27.22)	65 (36.11)
Total	83 (46.11)	97 (53.89)	180 (100.00)

APD: Acid Peptic Disease; FD: Functional Dyspepsia

**Table 4:** Endoscopic Findings of Acid Peptic Disease (APD) and Functional Dyspepsia (FD) in Relation to Risk Factors and Symptoms (n=180).

Group/Symptom	APD n (%)	FD n (%)	Total n (%)
**Smoking**			
Smokers	68(37.78)	13(7.22)	81(45.00)
Non-smokers	47(26.11)	52(28.89)	99(55.00)
**Alcohol Consumption**			
Consumers	62(34.44)	5(2.78)	67(37.22)
Non–consumers	53(29.44)	60(33.33)	113(62.78)
**Symptoms**			
Retrosternal burning sensation	113(62.78)	52(28.89)	165(91.67)
Upper abdominal pain	114(63.33)	4(2.22)	118(65.56)
Vomiting	30(16.67)	3(1.67)	33(18.33)
Early satiety	2(1.11)	64(35.56)	66(36.67)
Postprandial fullness	8(4.44)	64(35.56)	72(40.00)
**Duration of Symptoms**			
≥3 months	66(36.67)	40(22.22)	106(58.89)
1-3 months	49(27.22)	25(13.89)	74(41.11)
**Medication Use**			
Took medication for relief	13(7.22)	1(0.56)	14(7.78)

**Table 5:** Rapid Urease Test Results in Relation to Risk Factors and Symptoms (n=180).

Group/Symptom	Positive n(%)	Negative n(%)	Total n(%)
Overall	138(76.67)	42(23.33)	180(100.00)
**Smoking**			
Smokers	81(45.00)	0(0.00)	81(45.00)
Non-smokers	57(31.67)	42(23.33)	99(55.00)
Alcohol Consumption			
Consumers	66(36.67)	1(0.56)	67(37.22)
Non-consumers	72(40.00)	41(22.78)	113(62.78)
Duration of Symptoms			
>3 months	90(50.00)	16(8.89)	106(58.89)
1-3 months	48(26.67)	26(14.44)	74(41.11)

After performing the endoscopic procedure, 68 (84.00%) out of 81 (45.00%) participants who smoked had findings related to Acid Peptic Disease (APD), whereas 13 (16.00%) were classified as having Functional Dyspepsia (FD). Among the 99 (55.00%) nonsmokers, 47 (26.11%) had APD and 52 (28.89%) had FD . Among the 14 (7.78%) participants who reported medication use for symptom relief, 13 (7.22%) were diagnosed as having APD and 1 (0.56%) as FD (Table 4).

On rapid urease testing, 138 (76.67%) participants showed a positive result for H. pylori infection. All 81 (45.00%) participants who smoked had a positive rapid urease test. Among the 99 (55.00%) nonsmokers, 57 (31.67%) had a positive result. A positive rapid urease test was seen among 66 (36.67%) alcohol consumers 98.51% of consumer and 72 (40.00%) non-alcohol consumers (63.72%), (Table 5).

## DISCUSSION

Dyspepsia is a summary of upper gastrointestinal symptoms which includes upper abdominal pain, retrosternal burning sensation, early satiety, vomiting and postprandial fullness related to feeding.^1-2^

Several existing literatures have described their own findings related to the symptoms of dyspepsia. Majority have described upper abdominal or epigastric pain, retrosternal burning sensation, early satiety and postprandial fullness.^7-10^ In similar fashion, our study has interpreted that most patients presented to our institution with history of retrosternal burning 165 (91.67%), upper abdominal pain 118 (65.56%) followed by early satiety 66 (36.67%) and postprandial fullness 64 (35.56%) in that order. The majority of participants had these symptoms for more than 3 months.

*H. pylori* is a Gram-negative, spiral, urease producing, and highly pathogenic microaerophilic flagellated bacterium which causes several gastrointestinal disorders. One of which is acid peptic disease.^11^ Most of the dyspeptic patients in our study were diagnosed as having acid peptic disease 115 (63.89%), whereas the remaining participants were classified as having functional dyspepsia 65 (36.11%) (normal endoscopy findings) after performing an endoscopy.

Interestingly from the results shown above, we have interpreted that the endoscopic diagnosis of acid peptic disease and functional dyspepsia do not have such significance with the duration of symptoms. To clear on this statement from our results, 66 (62.26%) patients were diagnosed as APD out of 106 (58.89%) patients who had complained of the abovementioned symptoms for ≥3 months, compared to 49 (66.22%) patients diagnosed as APD out of 74 (41.11%) patients who had symptoms for 1-3 months.

A study conducted by Roshani, et al.^12^ revealed smoking as a major contributor and alcohol consumption as a cofactor for development and maintenance of peptic ulcer disease. Through our study we have tried to incorporate the effects of smoking and alcohol consumption among the dyspeptic patients. Smoking and alcohol consumption had more significant association with acid peptic disease than functional dyspepsia. This can be further strengthened by our result showing all patients who smoked showed a positive rapid urease test. However, among the non-consumers the cases of functional dyspepsia had also been proportionately increased with acid peptic disease.

Out of all the organic etiologies of dyspepsia, *H. pylori* infection is one of the most important and prevalent reported in the existing literature. A cross-sectional observational study done by Subedi, et al.^13^ reported a prevalence of 32.9% of *H pylori* infection among the dyspeptic patients. Similarly, Oung, et al.^14^ reported an overall prevalence of *H pylori* infection of 46% among the dyspeptic patients.

Our study also reported 138 (76.67%) individuals having a positive rapid urease test, and among those with positive results, 106 (76.81%) had accompanying endoscopic evidence of acid peptic disease. A positive rapid urease test of 138 (76.67%) was calculated from our study, which represented the prevalence of Helicobacter pylori infection. In stark contrast a descriptive cross-sectional study conducted by Shrestha, et al.^15^ (2012) in our institution reported the prevalence of *H pylori* of 50.47%, indicating a rise in the prevalence of *H.pylori* infection by 26.2% after 13 years. This might be due to several reasons including amount of smoking and patient’s diet. From our research results, we can also point toward a relation between a positive rapid urease test and the duration of symptoms. This means we calculated more positive test results in participants who had longer duration of symptoms (i.e. ≥ 3 months).

Our study has certain limitations that need to be acknowledged. Being conducted in a single tertiary care center, the findings may not truly reflect the situation in the wider community. Since we used total enumeration sampling without randomization, the possibility of selection bias cannot be ruled out. We relied mainly on rapid urease testing for detecting *H. pylori* infection, which, though widely used, may not be as accurate as other diagnostic tools such as histology or the urea breath test. Important lifestyle factors like diet, stress levels, and socioeconomic background were not studied in detail, even though they are known to influence both dyspeptic symptoms and *H. pylori* prevalence. Finally, as this was a cross-sectional study, it could only show associations and not establish a cause-and-effect relationship.

Future research should ideally be done across multiple centers with a larger number of participants, using random sampling to improve representativeness. Using a combination of diagnostic methods for *H. pylori* infection would help in achieving more reliable results. Longitudinal studies following patients over time could give clearer insights into how smoking, alcohol use, dietary habits, and infection with *H. pylori* contribute to the development of acid peptic disease. Further work may also explore the role of changing food habits, sanitation, and antibiotic resistance patterns of *H. pylori* in shaping the burden of disease in our setting.

## CONCLUSION

This study highlights that a large proportion of dyspeptic patients were diagnosed with acid peptic disease, and a high prevalence of Helicobacter pylori infection was observed using rapid urease testing compared to previous studies. Smoking and alcohol consumption were prevalent with acid peptic disease, while symptom duration was high in patients positive for H. pylori.

## Data Availability

The data are available from the corresponding author upon reasonable request.
